# Higher overall survival in metastatic pancreatic cancer: the impact of where and how treatment is delivered

**DOI:** 10.1590/S1679-45082015AO3303

**Published:** 2015

**Authors:** Pedro Luiz Serrano Usón, Monique Sedlmaier França, Heloisa Veasey Rodrigues, Antônio Luiz de Vasconcellos Macedo, Alberto Goldenberg, Oren Smaletz, Daniela Pezzutti Domingues Armentano, Sergio Daniel Simon, Rene Claudio Gansl

**Affiliations:** 1 Hospital Israelita Albert Einstein, São Paulo, SP, Brazil.; 2 Universidade Federal de São Paulo, São Paulo, SP, Brazil.

**Keywords:** Adenocarcinoma/drug therapy, Pancreatic neoplasms, Survivorship (Public Health)

## Abstract

**Objective:**

To determine the overall survival of patients with advanced pancreatic cancer and evaluate factors that impact prognosis in a private cancer center.

**Methods:**

Data from the Hospital Cancer Registry at Hospital Israelita Albert Einstein were retrospectively collected. The patients enrolled had metastatic cancer at diagnosis or earlier staging and subsequent recurrence. Cases of neuroendocrine tumors were excluded.

**Results:**

A total of 65 patients were evaluated, including 63 with adenocarcinoma. The median overall survival for patients in all stages was 20.7 months (95%CI: 15.6-25.7), while the overall survival of metastatic disease was 13.3 months. Among the 33 cases with stage IV cancer, there was no evidence of a statistically significant association between median survival and CA19-9 dosage (p=0.212), tumor location (p=0.482), first treatment performed (p=0.337), lymphovascular invasion (p=0.286), and age (p=0.152). However, the number of lines of chemotherapy was significantly associated with survival (log-rank p=0.013), with an estimated median survival of 10.2 months for patients who received up to two lines of treatment and 23.5 months for those receiving more than two lines of chemotherapy.

**Conclusion:**

The survival of patients treated was longer than that reported in the literature. The only statistically significant factor related to increased survival was higher number of lines of chemotherapy received. We believe that the higher socioeconomic status of patients surveyed in this study, as well as their greater access to treatment options, may have influenced their overall survival.

## INTRODUCTION

Pancreatic cancer is one of the most lethal malignancies worldwide, and ranks fourth in the total number of deaths related to cancer in patients of both genders. In 2013, the United States registered about 45 thousand new cases, and reported that the number of expected deaths was very similar to the number of new cases. The median overall survival at 5 years is between 2 and 6%.^(^ ^1^ ^)^

Adenocarcinoma of the pancreas is the most common type of pancreatic neoplasm, with all of its subtypes accounting for 85% of cases.^(^ ^1^ ^)^

Currently, curative treatment is only possible in cases of resectable disease and during the initial stages.^(^ ^2^ ^)^ Although complete surgical resection is the only potential curative approach of this disease, it can only be performed in 10 to 20% of patients, since most individuals present with advanced disease upon diagnosis.^(^ ^2^ ^,^ ^3^ ^)^ After surgical resection, 7 to 25% of patients have a 5-year survival rate,^(^ ^2^ ^)^ with better results in individuals who undergo curative resection (R0).^(^ ^4^ ^)^

Several factors are associated with poor prognosis after surgery with complete resection, such as levels of carbohydrate antigen 19-9 (CA19-9) at diagnosis, perineural invasion and tumor size.^(^ ^5^ ^)^

The median survival of patients with locally advanced disease is approximately 6 to 11 months.^(^ ^2^ ^)^ In cases of metastatic disease, chemotherapy increases overall survival and improves symptoms.^(^ ^6^ ^-^ ^8^ ^) ^The longest gain in median overall survival reported in the metastatic setting was 11.1 months, which was achieved with the combination of fluorouracil, leucovorin, irinotecan and oxaliplatin (FOLFIRINOX) treatment.^(^ ^9^ ^)^

Currently, easy access to all treatment modalities and to new combinations of chemotherapeutical drugs is considered an important factor that interferes in patient survival. Therefore, differences across services often lead to diverse prognoses for this disease.

## OBJECTIVE

This study aimed to determine the overall survival of patients with advanced pancreatic cancer in Hospital Israelita Albert Einstein, and evaluate possible factors that impact the prognosis of the disease.

## METHODS

Data were retrospectively collected from the Hospital Cancer Registry at Hospital Israelita Albert Einstein from the period between January 2007 and December 2013. Staging was determined based on the American Joint Committee on Cancer (AJCC)^(^ ^10^ ^) ^classification. Age, sex, tumor location, histopathological features, first treatment, surgery, date and site of recurrence or progression in case of metastatic disease, proposed treatment at relapse and number of lines of treatment received were obtained from patient’s medical records. The site of recurrence was obtained by imaging method chosen by the professional who attended the patient. The median survival was calculated for the period between the time of diagnosis and February 12, 2014, according to cancer stage. Finally, we calculated the median survival according to tumor location, lymphovascular and perineural invasion, level of CA19-9 marker at diagnosis, and number of lines of treatment, among others.

The median survival was estimated using the Kaplan-Meier method. The analysis of factors associated with survival of stage-IV patients was made by means of analysis of variance with log-rank tests and a Cox proportional hazards model.

Analyses were performed with the Statistical Package for the Social Science (SPSS) software version 17.0, and a 95% confidence interval (95%CI) and a 5% significance level were determined.

The study was approved by the Research Ethics Committee under register no. 687.990 and CAAE: 32349914.2.0000.0071

## RESULTS

A total of 146 cases of pancreatic cancer were identified, and patients in stages I, II and III who did not have tumor recurrences were excluded. Those suffering from neuroendocrine tumors were also excluded. Therefore, our final group consisted of 65 patients in all stages, including metastatic (stage IV). The final analysis included 43 deaths. The median age for all patients was 66 years and most were males (62%). The most common histological type was adenocarcinoma, representing 97% of cases. Half of the patients were stage IV at diagnosis and the majority had high CA19-9; only 15% of patients had CA19-9 within the normal range at diagnosis. As expected, the liver was the organ most frequently affected by metastasis among these patients. Chemotherapy alone was used as an initial treatment regimen in 50% of patients and most patients underwent only the first-line of treatment (53%), while another 12% underwent more than three lines of chemotherapy. Radiotherapy was used in 14% of cases with recurrence (nine patients). Other patient characteristics can be found in appendix 1.

Median overall survival for patients in all stages was 20.7 months (95%CI: 15.6-25.7) (Table 1).

**Table 1:** Survival according to cancer staging

Stage	n	Events (n)	Censored patients n (%)	Median survival (months)	95%CI	
					Lower limit	Upper limit
II	23	10	13 (56.5)	31.3	1.7	61.0
III	9	5	4 (44.4)	25.7	15.6	35.8
IV	33	28	5 (15.2)	13.3	6.3	20.2

Total	65	43	22 (33.8)	20.7	15.6	25.7

95%CI: 95% confidence interval.

We found no evidence of an association between patient death and serum CA19.9 (p=0.212), tumor location (p=0.482), first treatment performed (p=0.337), site of metastasis (p=0.197), lymphovascular invasion (p=0.286), age (p=0.152) and number of metastases (p=0.961). We were unable to evaluate the relationship between survival and stromal reaction, since no stage-IV patient in our group had this condition (Table 2).

**Table 2:** Analysis of factors associated with death

	n	Events (n)	Censored patients n (%)	Median survival (months)	95%CI		Log-rank p
					Lower limit	Upper limit	
Level of carbohydrate antigen 19-9							
<59 x ULN	13	11	2 (15.4)	17.5	7.2	27.8	0.212
≥59 x ULN	10	9	1 (10.0)	6.8	1.1	12.5	
Normal	10	8	2 (20.0)	13.3	6.1	20.4	
Pancreatic tumor site							
Head	19	15	4 (21.1)	15.6	6.1	25.0	0.482
Body	6	5	1 (16.7)	7.6	0.0	22.1	
Tail	8	8	0	10.2	9.2	11.3	
First treatment							
Surgery	2	2	0	7.6	--	--	0.337
Chemotherapy	30	25	5 (16.7)	15.6	4.0	27.1	
Chemo + radiotherapy	1	1	0	9.6	--	--	
Site of metastasis							
Liver	20	18	2 (10)	10.2	7.2	13.2	0.197
Other	13	10	3 (23.1)	15.6	6.1	25.0	
Lymphovascular invasion							
Yes	2	2	0	7.6	--	--	0.286
No	31	26	5 (16.1)	15.6	6.3	24.8	
Stromal reaction							
No	33	28	5 (15.2)	13.3	6.3	20.2	--
Number of chemotherapy lines							
Up to 2	26	22	4 (15.4)	10.2	6.2	14.2	0.013
More than 2	7	6	1 (14.3)	23.5	10.8	36.2	
Age							
<65	12	8	4 (33.3)	20.4	0.0	44.5	0.152
≥65	21	20	1 (4.8)	11.0	6.4	15.5	
Number of metastases							
Up to 2	28	23	5 (17.9)	11.0	6.2	15.8	0.961
More than 2	5	5	0	17.9	1.3	34.5	

95%CI: confidence interval 95%CI; ULN: upper limit of the normal range.

The only variable associated with survival in our group of patients was the number of lines of chemotherapy taken: the median survival was 10.2 months among patients with up to two lines of treatment and 23.5 months for patients with more than two lines of chemotherapy (log-rank p=0.013).

Relative to patients who underwent more than two lines of chemotherapy, the risk ratio estimated with the Cox model was 4.42 (95%CI: 1.25-15.55).

## DISCUSSION

The overall median survival of patients surveyed in this review was 20.7 months, which included patients in all stages of pancreatic cancer. The overall median survival for stage IV patients (metastatic disease at diagnosis) was 13.3 months.

According to the data validation of the 6th edition of staging of the AJCC, the median survival of patients in all stages of pancreatic adenocarcinoma (including patients undergoing pancreatectomy) was 12.6 months.^(^ ^10^ ^)^ In cases of stage IV at diagnosis, median survival was 2.5 months.

This difference in median survival may be related to many factors. It is known that treatment outcome in pancreatic cancer does not only depend on the chemotherapy regimen used, but also on the nature of the primary tumor and the surgery performed.^(^ ^2^ ^)^ In addition, most patients have multiple comorbidities that are also related to the epidemiology of cancer itself, such as smoking, obesity, diabetes and older age.^(^ ^11^ ^,^ ^12^ ^)^

Other variables known to influence treatment outcome include socioeconomic and performance status. A survey of more than 20 thousand cases of pancreatic cancer, conducted in California, revealed that race and socioeconomic background were related to differences in treatment and survival, with less favored economic groups having lower survival rates.^(^ ^13^ ^)^

The cases surveyed in our study were being treated in a private oncology referral center, with full access to modern chemotherapy regimens and procedures, such as prosthetics and surgery, and this may partly explain the difference in the results obtained.

Performance status is also a limiting factor for the onset and maintenance of systemic treatment. The vast majority of studies using first-line chemotherapy include only patients with good functional status, i.e., an Eastern Cooperative Oncology Group (ECOG) performance status 0-1 and a Karnofsky performance scale (KPS) >70.^(^ ^7^ ^,^ ^9^ ^,^ ^14^ ^)^

The only factor associated with better survival in our analysis was the number of lines of chemotherapy used, in which using more than two lines was associated with significantly better survival (23.5 months versus 10.2 months; p=0.013). We cannot exclude the possibility that patients who underwent more lines of treatment had better functional status at diagnosis.

The CA19-9 levels at diagnosis were used as an independent factor of poor prognosis in pancreatic cancer in several studies.^(^ ^15^ ^-^ ^17^ ^)^ However, in our study, we did not observe this pattern of association.

The same poor prognosis was associated with lymphovascular^(^ ^18^ ^,^ ^19^ ^)^ and perineural invasion,^(^ ^20^ ^-^ ^22^ ^)^ but we were not able to collect enough pathological data from our patients to evaluate these variables.

## CONCLUSION

In the group of patients studied at our private institution, we found better overall survival in patients with advanced scenario that reported in the literature. We did not find associations between prognosis and several variables that have previously been reported. One possible explanation could be the greater amount of resources available to these patients relative to the general population.

## References

[1] Siegel R, Naishadham D, Jemal A (2013). Cancer statistics, 2013. CA Cancer J Clin.

[2] Thomasset SC, Lobo DN (2010). Pancreatic cancer. Surgery (Oxford).

[3] Niederhuber JE, Brennan MF, Menck HR (1995). The national cancer database report on pancreatic cancer. Cancer.

[4] Wagner M, Redaelli C, Lietz M, Seiler CA, Friess H, Büchler MW (2004). Curative resection is the single most important factor determining outcome in patients with pancreatic adenocarcinoma. Br J Surg.

[5] Paik KY, Choi SH, Heo JS, Choi DW (2012). Analysis of liver metastasis after resection for pancreatic ductal adenocarcinoma. World J Gastrointest Oncol.

[6] Burris H, Moore MJ, Andersen J, Green MR, Rothenberg ML, Modiano MR, al et (1997). Improvements in survival and clinical benefit with gemcitabine as first-line therapy for patients with advanced pancreas cancer: a randomized trial. J Clin Oncol.

[7] Von Hoff DD, Ramanathan RK, Borad MJ, Laheru DA, Smith LS, Wood TE (2011). Gemcitabine plus nab-paclitaxel is an active regimen in patients with advanced pancreatic cancer: a phase I/II trial. J Clin Oncol.

[8] Moore MJ, Goldstein D, Hamm J, Figer A, Hecht JR, Gallinger S, Au HJ, Murawa P, Walde D, Wolff RA, Campos D, Lim R, Ding K, Clark G, Voskoglou-Nomikos T, Ptasynski M, Parulekar W, National Cancer Institute of Canada Clinical Trials Group (2007). Erlotinib plus gemcitabine compared with gemcitabine alone in patients with advanced pancreatic cancer: a phase III trial of the National Cancer Institute of Canada Clinical Trials Group. J Clin Oncol.

[9] Conroy T, Desseigne F, Ychou M, Bouché O, Guimbaud R, Bécouarn Y, Adenis A, Raoul JL, Gourgou-Bourgade S, de la Fouchardière C, Bennouna J, Bachet JB, Khemissa-Akouz F, Péré-Vergé D, Delbaldo C, Assenat E, Chauffert B, Michel P, Montoto-Grillot C, Ducreux M, Groupe Tumeurs Digestives of Unicancer, PRODIGE Intergroup (2011). FOLFIRINOX versus gemcitabine for metastatic pancreatic cancer. N Engl J Med.

[10] Bilimoria KY, Bentrem DJ, Ko CY, Ritchey J, Stewart AK, Winchester DP (2007). Validation of the 6th edition AJCC Pancreatic Cancer Staging System. Cancer.

[11] Raimondi S, Maisonneuve P, Lowenfels AB (2009). Epidemiology of pancreatic cancer: an overview. Nat Rev Gastroenterol Hepatol.

[12] Vincent A, Herman J, Schulick R, Hruban RH, Goggins M (2011). Pancreatic cancer. Lancet.

[13] Zell JA, Rhee JM, Ziogas A, Lipkin SM, Anton-Culver H (2007). Race, socioeconomic status, treatment, and survival time among pancreatic cancer cases in California. Cancer Epidemiol Biomarkers Prev.

[14] Yates JW, Chalmer B, McKegney FP (1980). Evaluation of patients with advanced cancer using the Karnofsky performance status. Cancer.

[15] Ni XG, Bai XF, Mao YL, Shao YF, Wu JX, Shan Y (2005). The clinical value of serum CEA, CA19-9, and CA242 in the diagnosis and prognosis of pancreatic cancer. Eur J Surg Oncol.

[16] Maisey NR, Norman AR, Hill A, Massey A, Oates J, Cunningham D (2005). CA19-9 as a prognostic factor in inoperable pancreatic cancer: the implication for clinical trials. Br J Cancer.

[17] Dusch N, Weiss C, Ströbel P, Kienle P, Post S, Niedergethmann M (2014). Factors predicting long-term survival following pancreatic resection for ductal adenocarcinoma of the pancreas: 40 years of experience. J Gastrointestl Surg.

[18] Chen JW, Bhandari M, Astill DS, Wilson TG, Kow L, Brooke-Smith M (2010). Predicting patient survival after pancreaticoduodenectomy for malignancy: histopathological criteria based on perineural infiltration and lymphovascular invasion. HPB (Oxford).

[19] Gao CT, Li HK, Li Q (2009). Factors influencing survival of patients with cancer of the pancreatic head after resection. Zhonghua Zhong Liu Za Zhi.

[20] Chatterjee D, Katz MH, Rashid A, Wang H, Iuga AC, Varadhachary GR (2012). Perineural and Intra-neural Invasion in posttherapy pancreaticoduodenectomy specimens predicts poor prognosis in patients with pancreatic ductal adenocarcinoma. Am J Surg Pathol.

[21] Garcea G, Dennison AR, Pattenden CJ, Neal CP, Sutton CD, Berry DP (2008). Survival following curative resection for pancreatic ductal adenocarcinoma. A systematic review of the literature. JOP.

[22] Chatterjee D, Rashid A, Wang H, Katz MH, Wolff RA, Varadhachary GR (2012). Tumor invasion of muscular vessels predicts poor prognosis in patients with pancreatic ductal adenocarcinoma who received neoadjuvant therapy and pancreaticoduodenectomy. Am J Surg Pathol.

