# Supplementary material for: Higher overall survival in metastatic pancreatic cancer: the impact of where and how treatment is delivered
Source: Einstein (Sao Paulo). 2015 Jul-Sep;13(3):347–51. doi: 10.1590/S1679-45082015AO3303 (PMC4943777; doi:10.1590/S1679-45082015AO3303)
Supplement: Translation

# Translation: Maior sobrevida global em pacientes com câncer pancreático metastático: o impacto de onde e como o tratamento é realizado

**Authors:** Pedro Luiz Serrano Usón, Monique Sedlmaier França, Heloisa Veasey Rodrigues, Antônio Luiz de Vasconcellos Macedo, Alberto Goldenberg, Oren Smaletz, Daniela Pezzutti Domingues Armentano, Sergio Daniel Simon, Rene Claudio Gansl

**Affiliations:** 1 Hospital Israelita Albert Einstein, São Paulo, SP, Brasil.; 2 Universidade Federal de São Paulo, São Paulo, SP, Brasil.

**Keywords:** Adenocarcinoma/quimioterapia, Neoplasias pancreáticas, Sobrevida (Saúde Pública)

## Abstract

**Objetivo:**

Determinar a sobrevida global dos pacientes com câncer pancreático avançado e avaliar fatores com impacto prognóstico em um centro de câncer privado.

**Métodos:**

Foram coletados retrospectivamente os dados do Registro de Câncer do Hospital Israelita Albert Einstein. Os pacientes incluídos apresentaram câncer metastático ao diagnóstico ou em estádio mais precoce com recorrência subsequente. Os casos de tumores neuroendócrinos foram excluídos.

**Resultados:**

Foram avaliados 65 pacientes, incluindo 63 com adenocarcinoma. A sobrevida global mediana dos pacientes em todos os estádios foi 20,7 meses (IC95%: 15,6-25,7), enquanto a sobrevida global de doença metastática foi de 13,3 meses. Entre os 33 casos com câncer em estádio IV, não houve evidência de associação estatisticamente significativa entre a sobrevida mediana e CA19-9 ao diagnóstico (p=0,212), localização do tumor (p=0,482), primeiro tratamento realizado (p=0,337), invasão vasculo-linfática (p=0,286) e idade (p=0,152). No entanto, o número de linhas de quimioterapia foi significativamente associado com a sobrevida (*log-rank* p=0,013), com uma sobrevida mediana estimada de 10,2 meses para os pacientes que receberam até duas linhas de tratamento e de 23,5 meses para os que receberam mais de duas linhas.

**Conclusão:**

A sobrevida dos pacientes tratados foi maior do que o relatado na literatura. O único fator estatisticamente significativo relacionado à maior sobrevida foi maior número de linhas de quimioterapia recebidas. Acreditamos que o nível socioeconômico dos pacientes pesquisados neste estudo, assim como seu maior acesso a opções de tratamento, pode ter influenciado em sua sobrevivência global.

## INTRODUÇÃO

O câncer de pâncreas é uma das neoplasias mais letais em todo o mundo e ocupa o quarto lugar em número total de mortes relacionadas ao câncer, em pacientes de ambos os sexos. Em 2013, os Estados Unidos registraram cerca de 45 mil casos novos e relataram que o número de mortes esperadas era bem próximo ao número de casos novos. A sobrevida global mediana em 5 anos é de 2 a 6%.^(^ ^1^ ^)^

O adenocarcinoma do pâncreas é o tipo mais comum de tumor pancreático e, com todos seus subtipos, representa 85% dos casos.^(^ ^1^ ^)^

Atualmente, o tratamento curativo só é possível nos casos de doença ressecável e nas fases iniciais.^(^ ^2^ ^)^ Embora a ressecção cirúrgica completa seja a única abordagem potencialmente curativa da doença, só pode ser realizada em 10 a 20% dos pacientes, já que a maioria dos pacientes apresenta doença avançada no momento do diagnóstico.^(^ ^2^ ^,^ ^3^ ^)^ Após a ressecção cirúrgica, 7 a 25% dos pacientes têm uma taxa de sobrevida de 5 anos,^(^ ^2^ ^)^ com melhores resultados para os pacientes que se submetem à ressecção curativa (R0).^(^ ^4^ ^)^

Diversos fatores estão associados a um mau prognóstico após a cirurgia com ressecção completa, como níveis de antígeno carboidrato 19-9 (CA19-9) no momento do diagnóstico, invasão perineural e tamanho do tumor.^(^ ^5^ ^)^

A sobrevida mediana de pacientes com doença localmente avançada é de aproximadamente 6 a 11 meses.^(^ ^2^ ^)^ Em casos de doença metastática, a quimioterapia aumenta a sobrevida global e melhora os sintomas.^(^ ^6^ ^-^ ^8^ ^) ^O maior ganho na sobrevida global mediana relatado no contexto metastático foi de 11,1 meses, que foi conseguido com a combinação de fluorouracil, leucovorina, irinotecano e oxaliplatina (FOLFIRINOX).^(^ ^9^ ^)^

Atualmente, o acesso fácil a todas as modalidades de tratamento e a novas combinações de quimioterápicos é considerado um fator importante, que afeta a sobrevida do paciente. Assim, as diferenças entre os serviços muitas vezes levam a diferenças de prognóstico para essa doença.

## OBJETIVO

Este estudo teve como objetivo determinar a sobrevida global dos pacientes com câncer pancreático avançado no Hospital Israelita Albert Einstein, e avaliar possíveis fatores que influenciam no prognóstico da doença.

## MÉTODOS

Os dados foram coletados retrospectivamente no Registro Hospitalar de Câncer do Hospital Israelita Albert Einstein, do período janeiro de 2007 a dezembro de 2013. O estadiamento foi determinado com base na classificação do *American Joint Committee on Cancer* (AJCC).^(^ ^10^ ^) ^Idade, sexo, localização do tumor, características histopatológicas, primeiro tratamento, cirurgia, data e local de recorrência ou progressão em caso de doença metastática, tratamento proposto na recidiva e número de linhas de tratamento recebido foram obtidos nos prontuários médicos. O local de recorrência foi obtido pelo método de imagem escolhido pelo profissional que atendeu o paciente. Calculou-se, então, a sobrevida mediana no período entre a data do diagnóstico até 12 de fevereiro de 2014, de acordo com o estádio do câncer. Finalmente, calculou-se a sobrevida mediana de acordo com a localização do tumor, a invasão linfovascular e perineural, o nível do marcador CA19-9 no diagnóstico, e o número de linhas de tratamento, entre outros.

A sobrevida mediana foi estimada pelo método de Kaplan-Meier. A verificação dos fatores associados à sobrevida dos pacientes em estádio IV foi feita por meio de uma análise de variância com os testes de *log-rank* e um modelo de riscos proporcionais de Cox.

As análises foram realizadas com o pacote estatístico *Statistical Package for the Social Science* (SPSS) versão 17.0, e estabeleceram-se um intervalo de confiança de 95% (IC95%) e um nível de significância de 5%.

O estudo foi aprovado pelos Comitês de Ética em Pesquisa sob o registo 687.990 e o CAAE: 32349914. 2.0000.0071

## RESULTADOS

Foram identificados 146 casos de câncer de pâncreas, dos quais foram excluídos os pacientes nos estádios I, II e III que não tiveram recorrência dos tumores. Os tumores neuroendócrinos também foram excluídos. Assim, nosso grupo final foi composto por 65 pacientes em todos os estádios, inclusive o metastático (estádio IV). A análise final incluiu 43 óbitos. A idade mediana para todos os pacientes foi 66 anos, e a maioria era do sexo masculino (62%). O tipo histológico mais comum foi o adenocarcinoma, representando 97% dos casos. Metade dos pacientes estavam no estádio IV no momento do diagnóstico e a maioria apresentava CA19-9 elevado; apenas 15% dos pacientes apresentavam CA19-9 na faixa da normalidade no momento do diagnóstico. Conforme esperado, o fígado foi o órgão mais frequentemente acometido por metástases entre esses pacientes. A quimioterapia isolada foi utilizada como regime de tratamento inicial em 50% dos indivíduos e a maioria dos pacientes foi submetida somente à primeira linha de tratamento (53%), ao passo que outros 12% foram submetidos a mais de três linhas de quimioterapia. A radioterapia foi utilizada em 14% dos casos com recorrência (nove pacientes). Outras características dos pacientes encontram-se no anexo 1.

A sobrevida mediana global para os pacientes em todos os estádios foi 20,7 meses (IC95%: 15,6-25,7). (Tabela 1).

**Tabela 1:** Sobrevida por estádio de câncer

Estádio	n	Eventos (n)	Pacientes censurados n (%)	Sobrevida mediana (meses)	IC 95%	
					Limite inferior	Limite superior
II	23	10	13 (56,5)	31,3	1,7	61,0
III	9	5	4 (44,4)	25,7	15,6	35,8
IV	33	28	5 (15,2)	13,3	6,3	20,2
Total	65	43	22 (33,8)	20,7	15,6	25,7

IC95%: intervalo de confiança 95%.

Não encontramos nenhuma evidência de alguma associação entre óbito do paciente e CA19-9 sérico (p=0,212), localização do tumor (p=0,482), primeiro tratamento realizado (p=0,337), local de metástase (p=0,197), invasão linfática (p=0,286), idade (p=0,152) e número de metástases (p=0,961). Não fomos capazes de avaliar a relação existente entre a sobrevida e a reação estromal, uma vez que nenhum paciente em estádio IV do nosso grupo apresentava essa condição (Tabela 2).

**Tabela 2:** Análise dos fatores associados a óbito

	n	Eventos (n)	Pacientes censurados n (%)	Sobrevida mediana (meses)	IC95%		*Log-rank* p
					Limite inferior	Limite superior	
Nível de antígeno carboidrato 19-9							
<59 x LSN	13	11	2 (15,4)	17,5	7,2	27,8	0,212
≥59 x LSN	10	9	1 (10,0)	6,8	1,1	12,5	
Normal	10	8	2 (20,0)	13,3	6,1	20,4	
Localização do tumor de pâncreas							
Cabeça	19	15	4 (21,1)	15,6	6,1	25,0	0,482
Corpo	6	5	1 (16,7)	7,6	0,0	22,1	
Cauda	8	8	0	10,2	9,2	11,3	
Primeiro tratamento							
Cirurgia	2	2	0	7,6	--	--	0,337
Quimioterapia	30	25	5 (16,7)	15,6	4,0	27,1	
Quimio + radioterapia	1	1	0	9,6	--	--	
Local das metástases							
Fígado	20	18	2 (10)	10,2	7,2	13,2	0,197
Outro	13	10	3 (23,1)	15,6	6,1	25,0	
Invasão linfovascular							
Sim	2	2	0	7,6	--	--	0,286
Não	31	26	5 (16,1)	15,6	6,3	24,8	
Reação estromal							
Não	33	28	5 (15,2)	13,3	6,3	20,2	--
Número de linhas de quimioterapia							
Até 2	26	22	4 (15,4)	10,2	6,2	14,2	0,013
Mais de 2	7	6	1 (14,3)	23,5	10,8	36,2	
Idade (anos)							
<65	12	8	4 (33,3)	20,4	0,0	44,5	0,152
≥65	21	20	1 (4,8)	11,0	6,4	15,5	
Número de metástases							
Até 2	28	23	5 (17,9)	11,0	6,2	15,8	0,961
Mais de 2	5	5	0	17,9	1,3	34,5	

IC95%: intervalo de confiança de 95%; LNS: limite superior da normalidade.

A única variável associada à sobrevida em nosso grupo de pacientes foi o número de linhas de quimioterapia recebidas: a sobrevida mediana foi de 10,2 meses entre os pacientes com até duas linhas de tratamento e de 23,5 meses para os pacientes com mais de duas linhas de quimioterapia (*log-rank* p=0,013).

Em relação aos pacientes que foram submetidos a mais de duas linhas de quimioterapia, a taxa de risco estimada com o modelo de Cox foi 4,42 (IC95%: 1,25-15,55).

## DISCUSSÃO

A sobrevida mediana dos pacientes pesquisados nesta revisão foi de 20,7 meses, incluindo pacientes em todos os estádios de câncer de pâncreas. A sobrevida mediana global para pacientes estádio IV (doença metastática no momento do diagnóstico) foi de 13,3 meses.

De acordo com a validação de dados da sexta edição do estadiamento da AJCC, a sobrevida mediana dos pacientes em todas as fases do adenocarcinoma do pâncreas (incluindo pacientes submetidos à pancreatectomia) foi 12,6 meses.^(^ ^10^ ^)^ Em casos de estádio IV no momento do diagnóstico, a sobrevida mediana foi 2,5 meses.

Essa diferença na sobrevida mediana pode estar relacionada a vários fatores. Sabe-se que o resultado do tratamento no câncer pancreático não depende apenas do regime de quimioterapia utilizado, mas também da natureza do tumor primário e da cirurgia realizada.^(^ ^2^ ^)^ Além disso, a maioria dos pacientes tem múltiplas comorbidades, que também estão relacionadas com a epidemiologia do câncer em si, como tabagismo, obesidade, diabetes e idade avançada.^(^ ^11^ ^,^ ^12^ ^)^

Outras variáveis que influenciam no resultado do tratamento incluem condição socioeconômica e *performance status*. Uma pesquisa de mais de 20 mil casos de câncer de pâncreas, realizada na Califórnia, revelou que raça e nível socioeconômico estavam relacionados a diferenças no tratamento e na sobrevida, com menores taxas de sobrevida nos grupos economicamente menos favorecidos.^(^ ^13^ ^)^

Os casos pesquisados em nosso estudo estavam sendo tratados em um centro oncológico de referência da rede privada, com acesso total aos regimes de quimioterapia e procedimentos modernos, como próteses e cirurgia, e isso pode explicar em parte a diferença nos resultados obtidos.

O *performance status* também é um fator limitante para o início e a manutenção do tratamento sistêmico. A maioria dos estudos que utilizaram quimioterapia de primeira linha incluiu apenas os pacientes com bom estado funcional, ou seja, um *performance status* de 0-1, na escala do *Eastern Cooperative Oncology Group* (ECOG), e *performance status* na escala de Karnofsky (KPS) >70.^(^ ^7^ ^,^ ^9^ ^,^ ^14^ ^)^

O único fator associado a uma maior sobrevida em nossa análise foi o número de linhas de quimioterapia utilizadas, sendo que o uso de mais de duas linhas estava associado a uma sobrevida significativamente melhor (23,5 meses *versus* 10,2 meses; p=0,013). Não podemos excluir a possibilidade de os pacientes submetidos a mais linhas de tratamento terem apresentado melhor estado funcional no momento do diagnóstico.

Os níveis de CA19-9 no momento do diagnóstico foram utilizados em vários estudos como um fator independente de pior prognóstico no câncer de pâncreas.^(^ ^15^ ^-^ ^17^ ^)^ No entanto, em nosso estudo, não observamos esse padrão de associação.

O mesmo prognóstico reservado foi associado à invasão linfovascular^(^ ^18^ ^,^ ^19^ ^)^ e perineural,^(^ ^20^ ^-^ ^22^ ^)^ mas não fomos capazes de coletar dados anatomopatológicos suficientes dos nossos pacientes para avaliar essas variáveis.

## CONCLUSÃO

No grupo de pacientes estudados em nossa instituição privada, observamos melhor sobrevida global nos pacientes em estádio avançado da doença do que a relatada na literatura. Não se encontrou associação entre prognóstico e diversas variáveis que foram previamente relatadas. Uma possível explicação poderia ser o maior número de recursos disponíveis para esses pacientes em relação à população em geral.
